# An Account of Three Cases, Viz. 1. of the Extraction and Depression of the Cataract in the Same Patient; 2. of an Encysted Hydrocele of the Tunica Communis of the Spermatic Chord, Communicating with the Tunica Vaginalis Testis; and 3. of an Amputation below the Knee, the Event of Which Shows That the Advantages of the Union by the First Intention, after Such an Operation, Are as Easily Obtained in the Leg as in the Thigh

**Published:** 1788

**Authors:** Richard Sparrow

**Affiliations:** one of the Surgeons of the Charitable Infirmary at Dublin


### THE LONDON MEDICAL JOURNAL.

I. *An Account of three Cases, viz.* 1. *of the Extraction and Depression of the Cataract in the same Patient*; 2. *of an encysted Hydrocele of the Tunica communis of the spermatic Chord communicating with the Tunica Vaginalis Testis*; *and* 3. *of an Amputation below the Knee, the Event of which shews that the Advantages of the Union by the first Intention, after such an Operation, are as easily obtained in the Leg as in the Thigh: by Mr.* Richard Sparrow, *one of the Surgeons of the Charitable Infirmary at Dublin. Communicated in a Letter to* William Lister, *M. D. Physician in London, and by him to Dr.* Simmons.

### CASE I.

*Extraction and Depression of the Cataract in the same Patient.*

ABIGAIL CREMOR, aged fifty years, came to the Charitable Infirmary in Dublin, with a cataract formed in each eye. That in the right eye was of four months, and that in the left of three years ſtanding. During their progreſs ſhe had been ſubject, at times, to pain in her head; and viſion was now ſo totally loſt, that it was only a very ſtrong light ſhe could diſtinguiſh from darkneſs: but the power of contraction and dilatation of the pupils was perfect.

The appearances being exactly ſimilar in both eyes, I determined to try the comparative merits of couching and extraction in a caſe which appeared to be ſo fair for the purpoſe. Accordingly, on the 25th of October, 1787, I extracted the lens from the left, and depreſſed it in the right eye. In the latter it adhered to the iris, ſo as to alter the ſhape of the pupil whilſt under the needle, and require repeated preſſure to prevent it from riſing again. Both the operations were performed fairly, and without accident; but the patient complained more of the extraction than of the depreſſion. The eyes were covered with bits of fine lint ſpread with ſaturnine ointment, and over theſe was placed a compreſs dipped in weak vegeto-mineral water. Blood was drawn from the jugular, and opiates, in ſmall doſes, were adminiſtered internally; an antiphlogiſtic plan of treatment was ſtrictly followed; lowed; and on the fifth day the bandage becoming loofe on the couched eye, I opened it. There was very little inflammation, and the patient could fee much light with it. As fhe remained fo free from pain, on the eighth day I examined the eye from which the lens had been extracted, and found the wound of the cornea united; the pupil much dilated, and irregular; the eye nearly full, with a good deal of inflammation, and unable to bear the light.

On the eleventh day the inflammation was totally gone from the couched eye, and fhe could fee objects with it. The inflammation of the other eye was alfo abated; the pupil, however, was ftill dilated, though with lefs irregularity, and fhe could fee light and objects with it, but not diftinctly.

On the twenty-fecond day, the couched eye was evidently getting ftronger, and there was only a little inflammation in the other; but the pupil of this eye was ftill dilated. She was now directed to wafh her eyes with cold water, and continued to mend till December 4th (the fortieth day after the operation) when fhe was difcharged from the hofpital with her eyes in the following ftate, viz.—The pupil of the couched eye fmaller than natural, and with very little little power of contraction, or dilatation; the vision of it imperfect, having the sensation of motes constantly flying before it, so as to impede, in some degree, the sight of the other, although the pupil was perfectly clear: the pupil of the other eye larger than natural, somewhat irregular and with little contractile power; the cicatrix of the cornea a little opake; but the vision so good, as to enable her to know all her former acquaintances, and to do many of the ordinary offices of life.

At the beginning of February, 1788, finding; for the first time since the operation, a slight return of the pain of her head, I judged it proper on account of this circumstance, as well as of her particular time of life, to make a pea issue in her arm which she thinks has done her service; and this day, March 2d, 1788, her eyes and vision are as follows:—The pupil of the couched eye is clear, and has recovered in a great degree its natural size, contraction, and dilatation; the vision is better, but still imperfect, having generally a sense of dust (as she says) flying before it, but upon the whole much mended; the pupil of the other eye is still larger than natural, but its irregularity is scarcely perceptible; the opacity of the cicatrix of the cornea is nearly gone, and and the viſion ſo perfect as to enable her to tell the hour on an eight-day clock without a glaſs, and in ſhort, to do, with eaſe, all the ordinary offices of life.

That extraction has ſucceeded in this caſe, beyond couching, is very evident; but whether this is to be attributed to the operation, or to the different degree of affection of the optic nerve, I ſhall not pretend to ſay; contenting myſelf with having given a faithful account of the caſe: it may, however, be obſerved, that in the couched eye viſion had been loſt only four months, whilſt, in the other, the patient had been deprived of it upwards of three years.

### CASE II.

*An encyſted Hydrocele of the Tunica Communis of the ſpermatic Chord, communicating with the Tunica Vaginalis Teſtis.*

SAMUEL MONK, a healthy looking man, aged thirty-ſix years, came to the Charitable Infirmary, on the 5th day of January, 1788, complaining of a tumour that extended from the groin to the bottom of the ſcrotum, a little below the middle of which there appeared an indentation, marking, as it were, the commencement of tunica vaginalis teſtis. Upon examination, a fluid was clearly to be felt in the tunica communis of the chord, and the teſticle ſeemed enlarged; but whether ſurrounded by a fluid, or not, was doubtful.

The account the man gave of his caſe was, that about a year before this, by a fall, he had hurt the ſcrotum and teſticle conſiderably; that the teſticle had continued to be ſwelled, but with no pain, till the beginning of November laſt, when he was thrown from a horſe, the immediate effect of which was a ſwelling, in the upper part of the ſcrotum, that had continued to increaſe, with much pain at times, up to the groin.

As there was evidently a fluid in the upper part of the tumour, while the preſence of any in the tunica vaginalis teſtis was very doubtful, I determined to begin my intended operation above, and to act afterwards as circumſtances might point out. I, therefore, made an inciſion into the upper part of the tumour on the chord, and diſcharged ſome ounces of a clear fluid, which appeared to have been contained in one of the cells of the tunica communis. The tumour at the bottom of the ſcrotum now appearing to be cauſed by the teſticle only, ſomewhat enlarged, but not hard, or unequal, it was, of courſe, left as it was; and having taken away a portion of the edges of the cyſt, I brought the lips of the wound into contact by ſuture, in order to procure an union by the firſt intention: but from the diviſion of ſome branches of an artery of the ſkin during the operation, an hemorrhage ſoon came on. I opened the ligatures to try to diſcover the veſſels from which it proceeded, but they had retracted within the cellular membrane. Upon preſſing the teſticle, accidentally, I obſerved a ſmall quantity of a clear fluid coming through the wound. This led me to examine more attentively, and I diſcovered, at the poſterior, inferior part of the cyſt, an opening, large enough to admit the end of my little finger, and communicating with the tunica vaginalis teſtis. I now diſtinctly felt the teſticle, which, as I juſt now obſerved, was enlarged, but neither hard, nor unequal; I therefore preſſed out ſome coagulated blood, which had inſinuated itſelf into the cavity, and to ſtop the bleeding, applied lint dipped in oil of turpentine: but ſome hours elapſed before the hemorrhage could be reſtrained, notwithſtanding this application was aſſiſted by conſiderable preſſure on the part.

On the third day, when I removed the dreſſings, the wound appeared foul, and the ſcrotum was much ſwelled, and tenſe, though with little pain. I now directed the ſcrotum to be covered with a ſoft poultice, and an anodyne to be adminiſtered at night. By this treatment, in ten days, the ſcrotum was reduced to its natural ſize, and the teſticle was neither painful nor very hard. Two grains of calomel were now ordered to be given every night; and, on the 5th of February, the wound (the edges of which had been brought nearly into contact by means of ſticking plaſter) being perfectly healed, the patient was diſmiſſed from the hoſpital. The teſticle was then diminiſhed in ſize, and much ſofter.

What makes this caſe of hydrocele ſingular, is the communication between the cyſt, and the tunica vaginalis teſtis; and that with ſuch a communication exiſting, the fluid, by its own gravity, ſhould not have been collected in greater quantity in the latter. This is not eaſily to be accounted for. Poſſibly it may be explained by ſuppoſing the cavity of the tunica vaginalis to have been nearly filled by the enlarged teſticle, and that the communication had been made ſo ſhort a time previous to the operation, that the preſſure preſſure of the fluid had not been long enough continued to diſtend the tunica vaginalis ſufficiently to enable it to contain a greater quantity.

### CASE III.

*An Amputation below the Knee, the Event of which ſhows that the Advantages of Union by the firſt Intention, after ſuch an Operation, are as eaſily obtained in the Leg as in the Thigh.*

MARY DANIEL, aged twelve years, applied, at the Charitable Infirmary, for relief for an extenſive caries of the bones of the leg and foot, with eroſion of the ligaments of the ancle joint. Hectic ſymptoms, increaſing rapidly, left her no reſource but in amputation. Accordingly, on the 26th of April, 1787, I performed the operation below the knee, beginning my inciſion through the ſkin (which was drawn up by an aſſiſtant), lower than uſual, and then, cutting obliquely upwards through the muſcles, I had a complete covering for the face of the ſtump. Having taken up the arteries, two in number, with a tenaculum and ligature, I brought the ſkin and muſcles together on the face of the ſtump, in a line from the tibia backwards, and ſecured them by ſlips of adheſive plaſter.

On

On the 30th of April, I removed the plaſters, and found the union complete, except where the ligatures protruded. One of theſe came away that day, and the other, on the day following.

On the 3d of May, the diſcharge was very ſmall; and, on the 9th, the wound was completely healed, excepting only a ſmall ſinus oppoſite the end of the tibia, which diſcharged about a tea-ſpoonful of matter daily.

On the 17th, this ſinus was contracted, and appeared to adhere to the end of the tibia. All diſcharge from it had ceaſed, and the ſtump being perfectly cicatrized, the patient was diſcharged from the hoſpital on that day (May the 17th) being the 21ſt from the operation. Her health was then mended in every reſpect.

This caſe is given to ſhow that the very great advantages of union by the firſt intention after amputation, are as eaſily obtained when this method is applied to the leg as to the thigh; which is contrary (I believe) to the general opinion.

*Dublin*,
March 2d, 1788.

